# Comparison of Blood Transfusion Rates Before and After Implementation of a Quality Improvement Initiative for Transfusion Safety and Appropriateness

**DOI:** 10.1001/jamanetworkopen.2022.52253

**Published:** 2023-01-23

**Authors:** Todd C. Lee, Nisha Almeida, Patricia Pelletier, Emily G. McDonald

**Affiliations:** 1 Division of Infectious Diseases, McGill University, Montreal, Quebec, Canada; 2 Clinical Practice Assessment Unit, McGill University Health Center, Montreal, Quebec, Canada; 3 Health Technology Assessment Unit, McGill University Health Center, Montreal, Quebec, Canada; 4 Division of Hematology, McGill University, Montreal, Quebec, Canada; 5 Division of General Internal Medicine, McGill University, Montreal, Quebec, Canada; 6 Centre for Outcomes Research and Evaluation, McGill University, Montreal, Quebec, Canada

## Abstract

This quality improvement study compares blood transfusion rates among patients at a single tertiary care hospital before and after the implementation of a transfusion training model completed by incoming resident physicians.

## Introduction

Blood products are a limited resource that should be used sparingly. Although packed red blood cells are lifesaving, there are known harms from transfusion, including circulatory overload, lung injury, infection, and blood type incompatibility reactions. Over the past 2 decades, landmark studies have demonstrated similar or improved outcomes with restrictive transfusion strategies for most conditions.^1^ In 2015, we piloted hemovigilance transfusion training in a medical teaching unit and demonstrated improved knowledge and modest improvements in blood use.^2^ In July 2019, the McGill University Health Center (MUHC) implemented a quality improvement intervention involving completion of the training module by incoming resident physicians. Herein, we compare the blood transfusion rates before and after implementation of this intervention.

## Methods

This quality improvement study followed the SQUIRE reporting guideline. The MUHC Research Ethics Board deemed this study exempt from review and waived the requirement of informed consent owing to the use of deidentified data.

In July and August of 2019 and 2020, MUHC resident physicians rotating in the upcoming academic year attended a 15-minute presentation on the importance of hemovigilance and were required to complete a previously described online training module.^2^ Residents who had already completed the training or who would not be transfusing blood during their training program were exempt. Trainees emailed the education office their completed training certificate or their self-reported exemption criterion. The only other cointervention was a prompt in the electronic medical record regarding transfusion thresholds, which had been in place since 2015.

Data were available for fiscal period 1 for 2017-2018 through fiscal period 8 for 2021-2022 (4 wk/period or 13 periods/y). Demographic data were not collected for age, sex, or race and ethnicity. Inpatient transfusions were obtained from the electronic data warehouse and were standardized per 1000 patient-days. The following units were excluded: psychiatry, emergency departments, intensive care units, birthing centers, outpatient surgery, operating rooms, and postanesthesia care units. Intervention outcomes were assessed graphically and with a segmented interrupted time series^3^ using the module ITSA^4^ in Stata, version 17 (StataCorp LLC). This module includes terms for the overall temporal trend, the introduction of the intervention, and the postintervention temporal trend. Results are reported as the change in rate per 1000 patient-days (intercept) and rate of change per 1000 patient-days per month (slope) with 95% CIs. All hypothesis tests were 2-sided using a *P* value of .05 for significance. A post hoc analysis included the onset of COVID-19 as an additional segment.

## Results

Of 1239 residents, 791 (64%) were nonexempt and completed the training. The monthly rates of transfusion are shown in the Figure. The intervention was associated with an immediate-level change of −1.96 transfusions per 1000 patient-days (95% CI, −3.77 to −0.15; *P* = .04) without statistically significant preintervention (−0.05 per 1000 patient-days per month [95% CI, −0.11 to 0.01; *P* = .11]) or postintervention (−0.03 per 1000 patient-days per month [95% CI, −0.10 to 0.04]; *P* = .45) temporal trends. Onset of COVID-19 was not associated with changes in transfusion rates.

**Figure.**
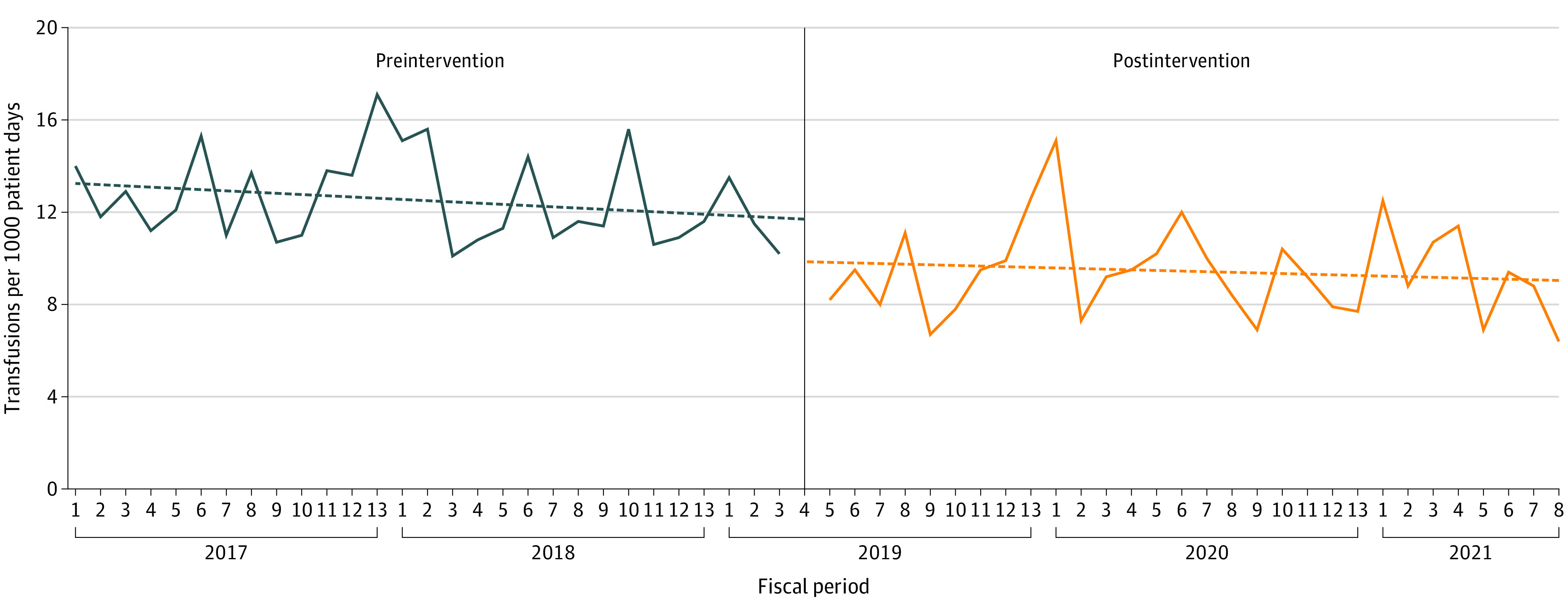
Transfusions per 1000 Patient-Days by Fiscal Period The vertical line indicates the start of the intervention.

## Discussion

The findings of this quality improvement study suggest that the implementation of a hemovigilance training module in our tertiary care hospital was associated with a decrease in the rate of blood transfusions. This change happened early, was detectable after accounting for overall temporal trends (assuming they were linear), and was large enough to be observed at the institutional level.

The major limitation of this study is the absence of a contemporaneous control. In addition, although some attending physicians volunteered to participate in the training, the intervention was mandatory only for trainees. If anything, we believe this would bias the results toward the null. Finally, we were not able to assess patient outcomes. Our findings suggest that standardized hemovigilance training could be a low-cost, low-barrier intervention to improve transfusion appropriateness and thus support the initiation of a multicenter randomized controlled trial.

## Notes

Supplement.
Data Sharing Statement
