# Supplementary material for: Comparison of Blood Transfusion Rates Before and After Implementation of a Quality Improvement Initiative for Transfusion Safety and Appropriateness
Source: JAMA Netw Open. 2023 Jan 23;6(1):e2252253. doi: 10.1001/jamanetworkopen.2022.52253 (PMC9871798; doi:10.1001/jamanetworkopen.2022.52253)
Supplement: Supplement. — Data Sharing Statement [file jamanetwopen-e2252253-s001.pdf]

Data Sharing Statement

Lee. Comparison of Blood Transfusion Rates Before and After Implementation of a Quality Improvement Initiative for Transfusion Safety and Appropriateness. *JAMA Netw Open*. Published January 23, 2023. doi:10.1001/jamanetworkopen.2022.52253

Data
Data available: No

Additional Information
Explanation for why data not available: We do not have individual patient data; only the aggregate data presented in the figure
